# Association of Heart Rate Variability With Silent Brain Infarcts in Patients With Atrial Fibrillation

**DOI:** 10.3389/fcvm.2021.684461

**Published:** 2021-05-21

**Authors:** Peter Hämmerle, Christian Eick, Sven Poli, Steffen Blum, Vincent Schlageter, Axel Bauer, Konstantinos D. Rizas, Ceylan Eken, Michael Coslovsky, Stefanie Aeschbacher, Philipp Krisai, Pascal Meyre, Jens Wuerfel, Tim Sinnecker, Jean-Marc Vesin, Jürg H. Beer, Giorgio Moschovitis, Leo H. Bonati, Christian Sticherling, David Conen, Stefan Osswald, Michael Kühne, Christine S. Zuern

**Affiliations:** ^1^Department of Cardiology, University Hospital Basel, Basel, Switzerland; ^2^Cardiovascular Research Institute Basel, University Hospital Basel, Basel, Switzerland; ^3^Department of Cardiology, University Hospital Tübingen, Tübingen, Germany; ^4^Department of Neurology & Stroke, Hertie Institute for Clinical Brain Research, Eberhard-Karls University Tübingen, Tübingen, Germany; ^5^University Clinic of Internal Medicine III, Cardiology and Angiology, Medical University of Innsbruck, Innsbruck, Austria; ^6^Medizinische Klinik und Poliklinik I, Munich University Clinic, Munich, Germany; ^7^German Center for Cardiovascular Research Partner Site, Munich Heart Alliance, Munich, Germany; ^8^Medical Image Analysis Center (MIAC) and Department of Biomedical Engineering, University of Basel, Basel, Switzerland; ^9^Department of Neurology and Stroke Center, University Hospital Basel, University of Basel, Basel, Switzerland; ^10^Institute of Electrical Engineneering, Swiss Federal Institute of Technology, Lausanne University Hospital, Lausanne, Switzerland; ^11^Department of Internal Medicine, Cantonal Hospital Baden, Aargau, Switzerland; ^12^Division of Cardiology, Ospedale Regionale di Lugano-Civico e Italiano, Lugano, Switzerland; ^13^Population Health Research Institute, McMaster University, Hamilton, ON, Canada

**Keywords:** atrial fibrillation, autonomic dysfunction, heart rate variability, silent brain infarct, HRV triangular index

## Abstract

**Purpose:** Silent brain infarcts (SBI) are frequently detected in patients with atrial fibrillation (AF), but it is unknown whether SBI are linked to autonomic dysfunction. We aimed to explore the association of autonomic dysfunction with SBI in AF patients.

**Methods:** 1,358 AF patients without prior stroke or TIA underwent brain MRI and 5-min resting ECG. We divided our cohort into AF patients who presented in sinus rhythm (SR-group, *n* = 816) or AF (AF-group, *n* = 542). HRV triangular index (HRVI), standard deviation of normal-to-normal intervals, mean heart rate, root mean square root of successive differences of normal-to-normal intervals, 5-min total power and power in the low frequency, high frequency and very low frequency range were calculated. Primary outcome was presence of SBI in the SR group, defined as large non-cortical or cortical infarcts. Secondary outcomes were SBI volumes and topography.

**Results:** Mean age was 72 ± 9 years, 27% were female. SBI were detected in 10.5% of the SR group and in 19.9% of the AF group (*p* < 0.001). HRVI <15 was the only HRV parameter associated with the presence of SBI after adjustment for clinical covariates in the SR group [odds ratio (OR) 1.67; 95% confidence interval (CI): 1.03–2.70; *p* = 0.037]. HRVI <15 was associated with larger brain infarct volumes [β (95% CI) −0.47 (−0.84; −0.09), *p* = 0.016] in the SR group and was more frequently observed in patients with right- than left-hemispheric SBI (*p* = 0.017).

**Conclusion:** Impaired HRVI is associated with SBI in AF patients. AF patients with autonomic dysfunction might undergo systematic brain MRI screening to initiate intensified medical treatment.

**Clinical Trials Gov Identifier:** NCT02105844.

## Introduction

Patients with atrial fibrillation (AF) have a high burden of silent brain infarcts (SBI) (1). SBI increase the risk of cognitive decline to a similar degree as overt strokes (1–3). Recently, it has been shown that silent large cortical and non-cortical infarcts (LNCCI) in AF patients were related to a 10-year age difference in cognitive performance (1). Furthermore, individuals with silent subcortical brain infarcts are at high risk for overt stroke (4). Therefore, the prevention and timely identification of SBI is a major public health concern. AF patients with SBI might benefit from an intensive control of vascular risk factors and dedicated neuropsychological treatment. However, systematic brain magnetic resonance imaging (bMRI) screening to detect SBI in patients with AF is not feasible due to reduced availability, costs and contraindications. An easily available tool that would allow for identification of AF patients at high risk for SBI would therefore be of high clinical value.

Overt strokes have been commonly associated with autonomic dysfunction (5–7). The functional status of the autonomic nervous system can be non-invasively assessed by the analysis of heart rate variability (HRV) (8). Parameters of HRV have been shown to predict overt stroke as well as complications and functionality of stroke survivors (9).

However, it is still unknown whether autonomic dysfunction is associated with SBI in AF patients. The primary objective of this analysis was to assess if impaired HRV is associated with presence of SBI in AF patients who were constantly in sinus rhythm (SR) at the time of the ECG recording (“SR group”). Secondary objectives were to (1) investigate the association between impairment of HRV and volumes of SBI and (2) analyze the association of HRV with topography of SBI. Exploratory analyses testing the association of HRV with the presence, volume and topography of SB were conducted in patients who were constantly in AF at the time of ECG recording (“AF group”).

## Materials and Methods

### Patient Population

The Swiss Atrial Fibrillation (Swiss-AF) cohort is an ongoing, multicenter prospective cohort study that enrolled 2,415 patients with AF at 14 study sites across Switzerland (10). Detailed information of the design and methodology including sample size calculations have been described elsewhere (ClinicalTrials.gov NCT02105844) (10). In brief, patients were eligible for Swiss-AF if they were aged ≥65 years and had a documented history of AF. A limited subset of patients aged 45–65 years was also enrolled. Main exclusion criteria for Swiss-AF were the presence of provoked, short and reversible AF episodes (e.g., after surgery), the inability to give informed consent as well as any acute illness within 4 weeks prior to enrolment.

**Figure 1:**
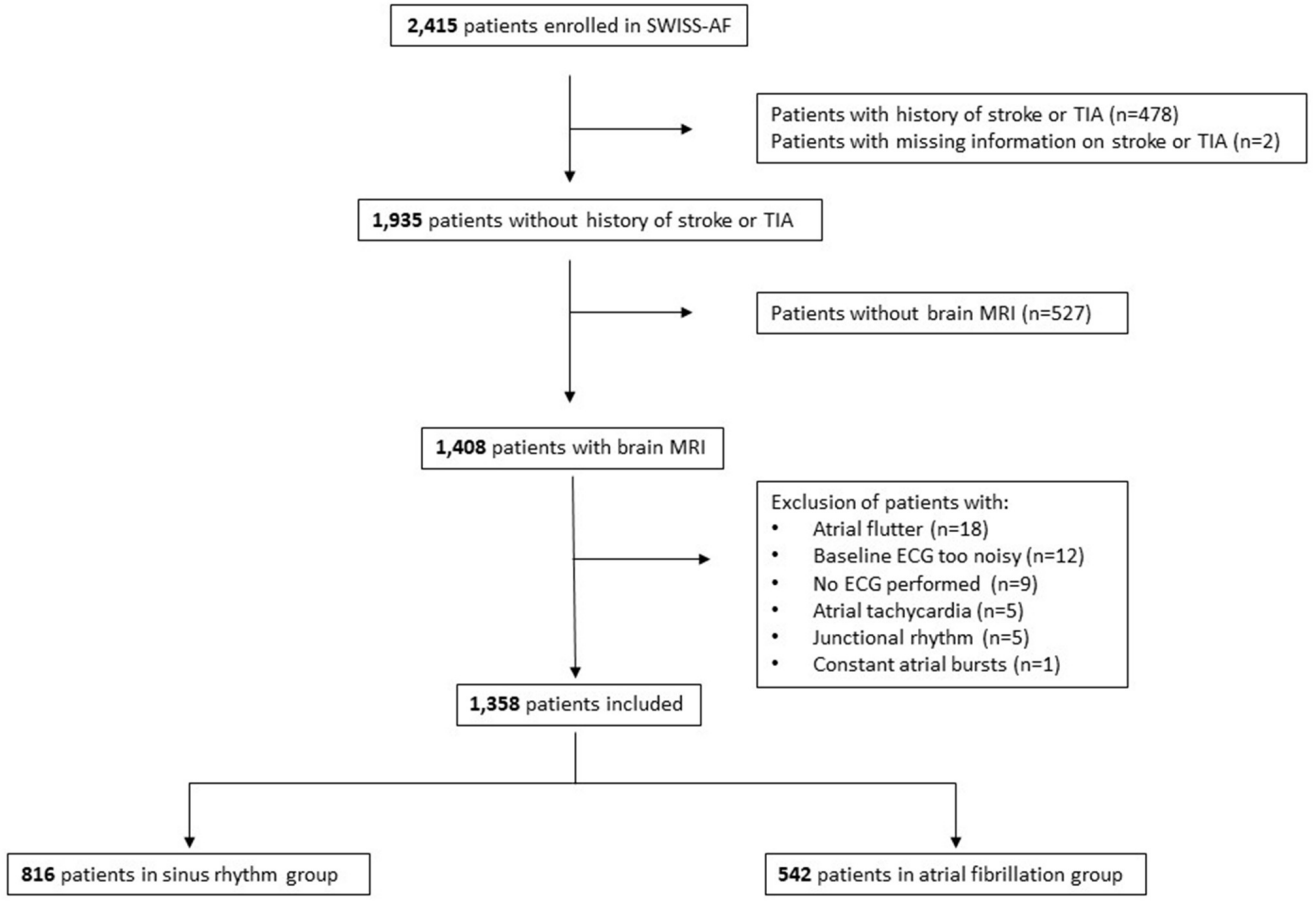
Flow chart of patient selection.

Of the 2,415 patients enrolled in Swiss-AF (Figure 1), we excluded 480 patients (19.9%) due to known and documented history of stroke or transient ischemic attack (TIA) or due to lack of respective information. From the remaining patients, we excluded 527 due to missing bMRI (mostly patients with cardiac devices or claustrophobia). Finally, we excluded 21 patients due to missing or low-quality baseline ECG, and 29 patients due to rhythm other than SR or AF. Thus, 1,358 patients remained for the present analysis. In order to analyse the association of HRV with SBI during SR and AF separately, the rhythm on the baseline resting ECG was used to allocate patients to two groups: the “SR- group” (*n* = 816) and the AF-group (*n* = 542). Of note, all 1,358 included patients are AF patients. The SR group includes patients with paroxysmal and persistent AF who were in SR at the time of the ECG. The AF group includes patients with paroxysmal, persistent and permanent AF (AF types defined according to ESC guidelines) (11). The study protocol has been approved by the local ethics committees, and all participants provided informed consent.

### Clinical Parameters

In order to obtain information on personal characteristics, prior medical history, comorbidities, medical and interventional treatment and other risk factors standardized case report forms were used by trained study personnel.

### ECG Recordings

Baseline resting ECG (CS-200 Excellence and CS-200 Touch, Schiller AG, Baar, Switzerland) comprised 16 leads and were recorded for at least 300 seconds. ECG were digitally saved on a central server with a sampling frequency of 1 kHz (signal bandwidth 0.04-387 Hz) and a resolution of 1μV/bit. We used an automated R-peak detection algorithm, based on a Shannon energy envelope estimator and a Hilbert transformation, as described elsewhere (12). The following parameters of HRV were calculated from the total recordings (115 ECG segments with excessive artifact burden from 98 patients were disregarded), according to previously published algorithms (8, 13): heart rate variability triangular index (HRVI), standard deviation of the normal-to-normal intervals (SDNN), mean heart rate (MHR) and root mean square root of successive differences of normal-to-normal intervals (rMSSD). Spectral analysis was performed to calculate the following frequency domain measures of HRV (8): 5-min total power, power in the low frequency range (LF, 0.04–0.15 Hz), in the high frequency range (HF, 0.15–0.4 Hz) and in the very low frequency range (VLF, ≤ 0.04 Hz).

### Brain Magnetic Resonance Imaging

The detailed methodology of bMRI analysis within the Swiss-AF cohort study has been described previously (1). In short, bMRI scans were acquired either on 1.5 T or a 3 T scanners with a standardized protocol defining an admissible range of image parameters at all study centers and analyses were performed in the neuroimaging core laboratory at Medical Image Analysis Centre, Basel, Switzerland. Blinded expert raters, who were unaware of the patients‘ characteristics, analyzed all bMRI scans and board-certified neuroradiologists verified the ratings. Brain infarcts were assessed on MPRAGE, FLAIR, and DWI based on lesion morphology consistent with acute or chronic ischemic infarction.

In more detail, large non-cortical infarcts were defined as hyperintense lesions on FLAIR >20 mm in diameter on axial sections not involving the cortex. These brain lesions had to be in line with an ischemia in the territory of a perforating arteriole located in the white matter, internal or external capsule, deep brain nuclei, thalamus, or brainstem (14). Cortical infarcts were hyperintense lesions on FLAIR involving the cortex regardless of their size (1). We combined large LNCCIs into one category. T2-weighted volumes of non-cortical and cortical infarcts were segmented and quantified by trained specialists in a validated process (software-supported threshold-based segmentation and edge-detection implemented in the Amira software package, Mercury Computer Systems Inc., Chelmsford, Massachusetts) to ensure consistent and reliable lesion and infarct size determination. SBI were defined as LNCCIs detected on bMRI, as all patients with known and documented history of stroke or TIA were a priori excluded from this study (Figure 1). Further, SBI were categorized into left-, right- and bi-hemispheric brain infarcts.

### Statistical Analysis

Analyses were performed based on a pre-determined statistical analysis plan approved internally by the Swiss-AF steering committee. The patients‘ clinical characteristics at baseline were stratified according to the baseline ECG rhythm (SR group vs. AF group). Categorical variables are expressed as counts (percentages) and were compared using the chi-square test. The distribution of continuous variables was checked by visual inspection of the histogram and by assessing skewness and kurtosis. Continuous variables are presented as mean ± standard deviation and are compared using Student's *t*-tests, or presented as median [interquartile range (IQR)] and compared using Mann-Whitney *U*-Test, as appropriate. Outcome analyses were performed in the SR group, whereas in the AF group, outcome analyses were only of exploratory nature. HRV parameters were used as main independent predictor variables in separate binary logistic regression models with the presence of LNCCI as the dependent variable. Study center was included as a random intercept to account for potential differences across study centers. To assess the association of HRV and volumes of SBI in patients who had at least one SBI, we built linear regression models for each predictor. First, we used crude models. Second, multivariable analyses were done by including a set of covariates determined via expert opinion and experience: age, sex, systolic blood pressure, and history of hypertension, history of diabetes, history of heart failure, prior myocardial infarction, prior major bleeding, prior pulmonary vein isolation (PVI) and intake of oral anticoagulation, antiarrhythmic drugs and beta-blockers. We prospectively dichotomized HRVI at 15 according to data of St George‘s Postinfarction Research Survey Programme and according to previously published data of our Swiss-AF cohort (8, 15), SDNN at 70 ms according to the ATRAMI cut-off (3), that was also used in the GISSI-2 study (16, 17), MHR at 80 bpm according to the ESC guidelines for the management of AF (11, 18) and rMSSD at 42 ms as published previously (19). Frequency domain measures of HRV were log-transformed. We examined whether the association of HRVI and presence of SBI depends on patient characteristics (age < median vs. age ≥ median, males vs. females, paroxysmal vs. persistent AF type, history of diabetes, history of myocardial infarction, intake of antiarrhythmic drugs, history of PVI) first by testing the interaction of HRVI with the relevant characteristic and then by fitting models again within the subgroup. Each interaction was entered to the full multivariable model separately in turn and tested, following with the relevant subgroup analyses with no interactions. Due to the skewed distribution, volumes of clinically SBI were log-transformed. Results are presented as odds ratio (OR) or beta-coefficients (β) with the corresponding 95% confidence intervals (CI). Statistical analyses were performed using SPSS Statistics for Windows, Version 25 (IBM Corp., Armonk, NY, USA) and SAS 9.4 (SAS Corporation, Cary, North Carolina, USA).

## Results

Baseline characteristics of the SR group (*n* = 816 patients) and the AF group (*n* = 542 patients) are presented in Table 1. Patients in the AF group were older (75 ± 8 vs. 70 ± 9 years) and were less often female (22 vs. 30%) and had higher rates of comorbidities, such as hypertension, diabetes and prior heart failure. Mean CHA_2_DS_2_-VASc score was 3.2 ± 1.4 in the AF group and 2.8 ± 3.9 in the SR group. The average duration of ECG recording was 300.2 ± 3.5 s in the SR group and 300.3 ± 5.1 s in the AF group. Time domain and frequency domain measures of HRV were higher in the AF group than in the SR group (Supplementary Table 1).

**Table 1:** Characteristics of the patients stratified by baseline rhythm.

**Characteristic**	**Sinus rhythm group (*n* = 816)**	**Atrial fibrillation group (*n* = 542)**	*p*-value^*^
Age, years	70 ± 9	75 ± 8	<0.001
Female sex (%)	242 (30)	118 (22)	0.001
Body mass index, kg/m^2^	27.2 ± 4.9	28.5 ± 5.0	<0.001
Systolic/diastolic blood pressure, mmHg	137 ± 18/78 ± 11	133 ± 18/80 ± 13	0.004/0.003
History of hypertension (%)	521 (64)	401 (74)	<0.001
History of diabetes mellitus (%)	100 (12)	99 (18)	0.002
Active and former smokers (%)	459 (56)	302 (56)	0.847
History of electrocardioversion (%)	325 (40)	200 (37)	0.278
History of pulmonary vein isolation (%)	300 (37)	38 (7)	<0.001
History of myocardial infarction (%)	92 (11)	87 (16)	0.011
History of percutaneous coronary intervention (%)	145 (18)	133 (25)	0.002
History of heart failure (%)	121 (15)	171 (32)	<0.001
History of chronic kidney disease (%)	102 (13)	127 (23)	<0.001
CHA_2_DS_2_-VASc score, points	2.8 ± 3.9	3.2 ± 1.4	<0.001
History of major bleeding (%)	34 (4)	37 (7)	0.031
Paroxysmal atrial fibrillation (%)	531 (65)	66 (12)	<0.001
Persistent atrial fibrillation (%)	285 (35)	167 (31)	0.115
Permanent atrial fibrillation (%)	0 (0)	309 (57)	–
Antiarrhythmic therapy (class Ic and III) (%)	261 (32)	143 (26)	0.027
Beta-blockers (%)	520 (64)	376 (69)	0.031
Non Vitamin K oral anticoagulants (%)	500 (61)	219 (40)	<0.001
Vitamin K antagonists (%)	196 (24)	293 (54)	<0.001

*Data are means ± SD or counts (percentages)*.

* *p-value compares sinus rhythm and atrial fibrillation groups. P-values were obtained from Student's t-tests for continuous variables and chi-square tests for categorical variables. CHA_2_DS_2_-VASc, congestive heart failure, hypertension, age ≥75 yeas (2 points), diabetes, prior stroke or TIA or thromboembolism (2 points), vascular disease, age 65 to 74 years, female sex; n, number*.

In the total study cohort (*n* = 1, 358), the prevalence of SBI was 14.3% (*n* = 194) and the median (IQR) volume was 531 (153–3,510) mm^3^. In the AF group, prevalence and median (IQR) volume of SBI was 19.9% (*n* = 108) and 747 (434–4,615) mm^3^. In the SR group, prevalence of SBI was 10.5% (*n* = 86) and median (IQR) volume was 354 (132–2,016) mm^3^, (*p* < 0.001 for both comparisons between the AF and SR group).

### Association of HRV With Presence and Volume of Silent Brain Infarcts in the SR Group

**Figure 2:**
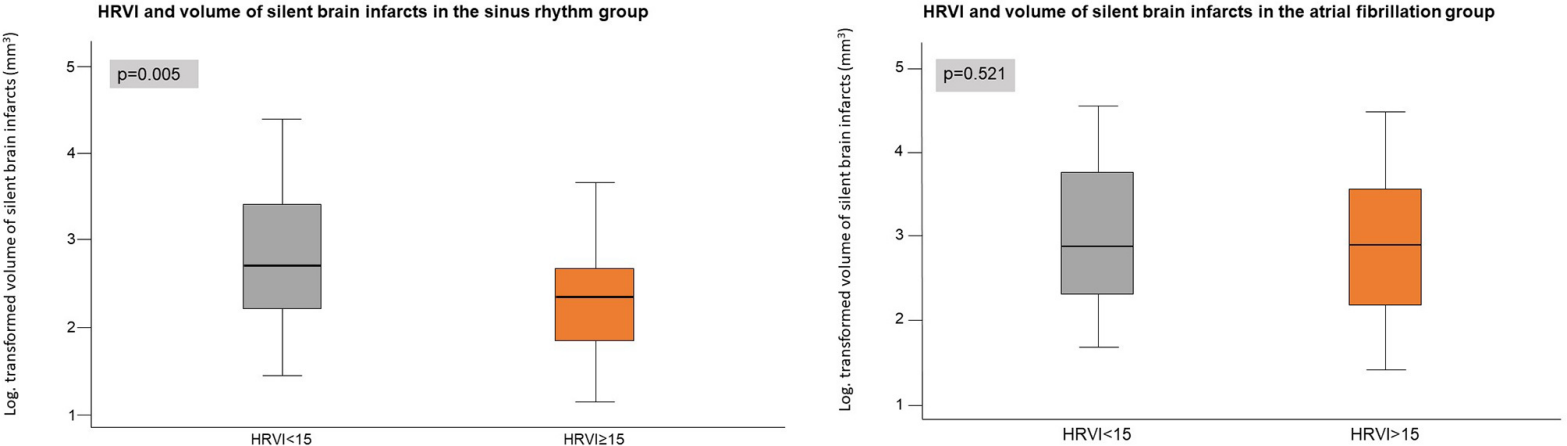
Volumes of silent brain infarcts in the sinus rhythm group **(left)** and in the atrial fibrillation group (**right**, exploratory analysis) stratified by heart rate variability triangular index (HRVI).

The univariable OR for the association of HRV parameters with presence of SBI are presented in Table 2. Only HRVI <15 was associated, yielding an OR of 1.69 (95% CI 1.05–2.70, *p* = 0.030). In the multivariable model, HRVI <15 remained the only HRV parameter associated with the presence of SBI (OR 1.67, 95% CI 1.03–2.70, *p* = 0.037). For HRVI and presence of SBI, no interactions were observed in any of the subgroups (Table 3). In multivariable adjusted linear regression models, HRVI was the only HRV parameter related to LNCCI volumes (Table 4), with HRVI <15 predicting higher SBI volume [β (95% CI) −0.47 (−0.84; −0.09), *p* = 0.016, Figure 2, left panel].

**Table 2:** Association of heart rate variability and presence of silent brain infarcts in the sinus rhythm group.

**HRV parameter**	**Univariable model OR (95% CI)**	*p*-value^*^	**Multivariable model OR (95% CI)**	*p*-value^*^
**Time domain measures**				
HRVI <15	1.69 (1.05–2.70)	0.030	1.67 (1.03–2.70)	0.037
SDNN <70ms	1.51 (0.93–2.43)	0.093	1.53 (0.90–2.39)	0.087
rMSSD <42ms	1.15 (0.73–1.83)	0.550	1.16 (0.72–1.86)	0.537
MHR>80bpm	1.00 (0.64–1.56)	0.991	1.02 (0.64–1.61)	0.934
**Frequency domain measures**^†^				
5-min total power	1.33 (0.97–1.82)	0.078	1.38 (1.00–1.90)	0.050
LF	1.29 (0.96–1.74)	0.090	1.34 (0.99–1.81)	0.057
HF	1.31 (0.95–1.80)	0.103	1.36 (0.98–1.90)	0.066
VLF	1.22 (0.93–1.61)	0.157	1.26 (0.94–1.67)	0.118

*Data are odds ratios (OR) (95% confidence intervals [CI])*.

* *p-values were based on logistic regression models*.

† *Frequency domain measures of HRV have been log-transformed. Study center was included as random intercept. Multivariable model was adjusted for age, sex, systolic blood pressure, history of hypertension, history of diabetes, history of heart failure, prior myocardial infarction, prior major bleeding, history of pulmonary vein isolation, intake of oral anticoagulation, antiarrhythmics and betablockers. HF, high frequency (0.15–0.4 Hz); HRV, heart rate variability; HRVI, heart rate variability triangular index; MHR, mean heart rate; LF, low frequency (0.04–0.15 Hz); OR, odds ratio; rMSSD, root mean square root of successive differences of normal-to-normal intervals; SDNN, standard deviation of the normal-to-normal intervals; VLF, very low frequency (≤ 0.04 Hz)*.

**Table 3:** Association of heart rate variability triangular index and silent brain infarcts: subgroup analyses in the sinus rhythm group.

**Subgroup**	**No. of events/n**	**Odds ratio**	**95% CI**	***p*-value**	**p-interaction**
**Age**					
Age < median	40/408	1.52	0.78–2.95	0.220	0.302
Age≥median	46/408	1.84	0.94–3.61	0.077	
**Sex**					
Male	66/574	1.48	0.87–2.52	0.146	0.132
Female	20/242	2.64	0.93–7.52	0.068	
**AF type**					
Paroxysmal	54/531	1.19	0.68–2.10	0.540	0.173
Persistent	32/285	3.77	1.41–10.1	0.008	
**History of diabetes**					
Yes	13/100	4.90	1.03–23.4	0.046	0.111
No	73/716	1.45	0.88–2.34	0.143	
**History of myocardial infarction**					
Yes	13/92	1.61	0.46–5.69	0.457	0.381
No	73/724	1.68	1.01–2.79	0.045	
**Intake of antiarrhythmic drugs (class Ic, II & III)**					
Yes	68/626	1.95	1.14–3.35	0.015	0.271
No	18/190	1.01	0.38–2.69	0.979	
**History of pulmonary vein isolation**					
Yes	35/300	1.30	0.63–2.66	0.475	0.917
No	51/516	2.05	1.09–3.84	0.026	

*Univariable analyses are presented. AF, atrial fibrillation; CI, confidence interval; No, number; n, number of patients included in the subgroup*.

**Table 4:** Association of heart rate variability and silent brain infarct volume in the sinus rhythm group.

**HRV parameter**	**Univariable model** ** β (95% CI)**	*p*-value^*^	**Multivariable model** ** β (95% CI)**	*p*-value^*^
**Time domain measures**				
HRVI <15	0.49 (0.14; 0.83)	0.007	−0.47 (−0.84; −0.09)	0.016
SDNN <70ms	0.30 (−0.06; 0.66)	0.103	0.24 (−0.15; 0.63)	0.222
rMSSD <42ms	0.11 (−0.25; 0.47)	0.550	0.01 (−0.39; 0.41)	0.946
MHR>80bpm	0.13 (−0.21; 0.47)	0.453	0.17 (−0.22; 0.55)	0.389
**Frequency domain measures**^†^				
5-min total power	0.19 (−0.06; 0.46)	0.140	0.24 (−0.02; 0.50)	0.065
LF	0.21 (−0.03; 0.44)	0.090	0.24 (−0.01; 0.49)	0.061
HF	0.06 (−0.18; 0.31)	0.603	0.12 (−0.15; 0.38)	0.380
VLF	0.12 (−0.10; 0.33)	0.286	0.17 (−0.06; 0.40)	0.149

*Data are beta-coefficients (β) [95% confidence intervals (CI)]. Brain infarct volumes were log-transformed*.

* *p-values were based on linear regression models*.

† *Frequency domain measures of HRV have been log-transformed. Study center was included as random intercept. Multivariable model was adjusted for age, sex, systolic blood pressure, history of hypertension, history of diabetes, history of heart failure, prior myocardial infarction, prior major bleeding, history of pulmonary vein isolation, intake of oral anticoagulation, antiarrhythmics and betablockers. HF, high frequency (0.15–0.4 Hz); HRV, heart rate variability; HRVI, heart rate variability triangular index; MHR, mean heart rate; LF, low frequency (0.04–0.15 Hz); rMSSD, root mean square root of successive differences of normal-to-normal intervals; SDNN, standard deviation of the normal-to-normal intervals; VLF, very low frequency (≤ 0.04 Hz)*.

### Association of HRV With Topography of Silent Brain Infarcts

**Figure 3:**
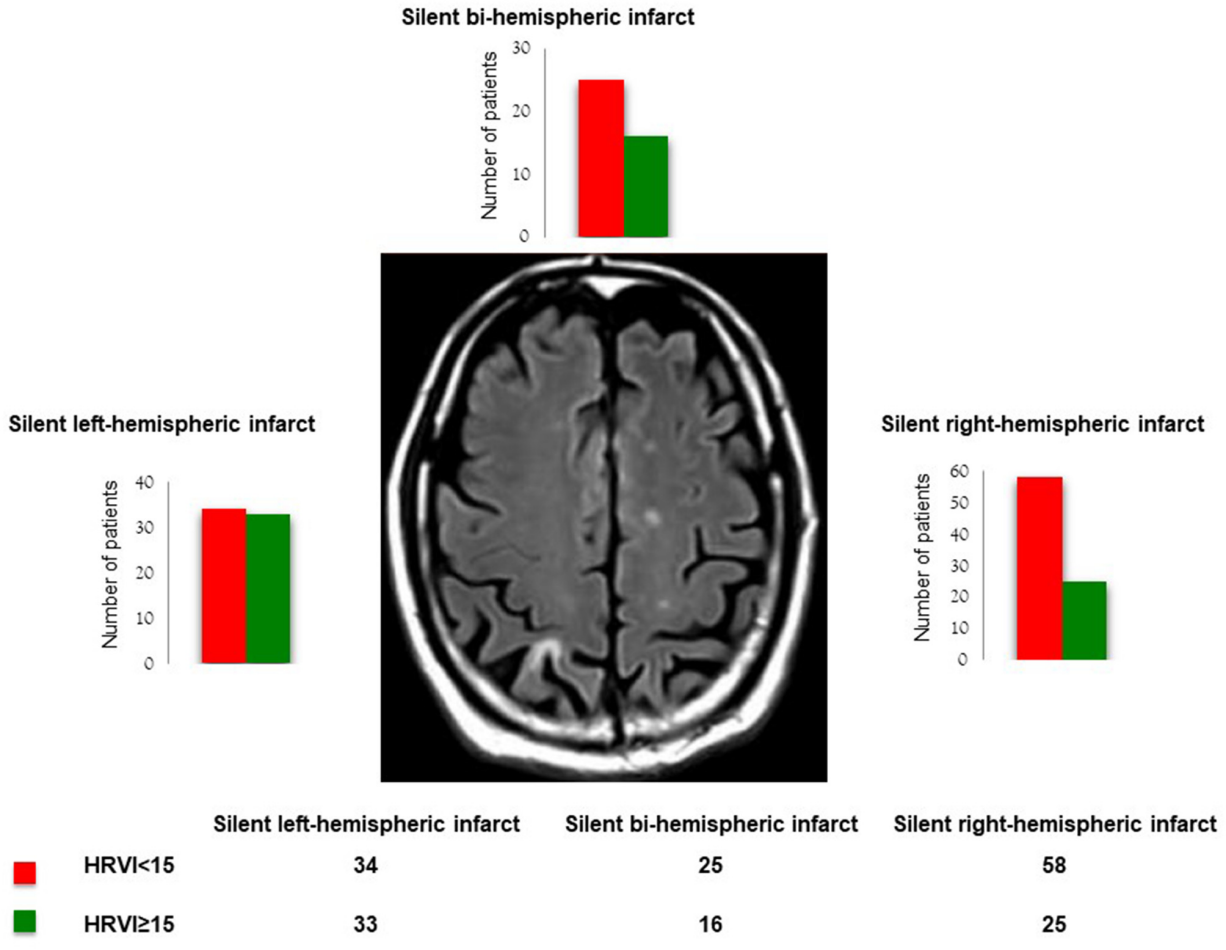
Impairment of heart rate variability triangular index (HRVI) according silent brain infarct topography.

Eighty three patients had right-hemispheric, 67 left-hemispheric and 41 bi-hemispheric brain infarcts (Figure 3). Three patients had a SBI in a localization, which could not be allocated to one hemisphere. In patients with right-hemispheric infarcts, HRVI was lower compared to patients with left-hemispheric SBI [median 13.6 (IQR 11.3–16.2) vs. 14.9 (12.1–20.0), *p* = 0.022], whereas SDNN, rMSSD, MHR, 5-min total power, LF, HF and VLF did not differ. All HRV parameters were similar in patients with right-hemispheric vs. bi-hemispheric infarcts and in patients with bi-hemispheric vs. left-hemispheric brain infarcts. Sixty nine point nine percentage of the patients with right-hemispheric SBI had an HRVI <15, in comparison to 50.7% of patients with left-hemispheric brain infarct (*p* = 0.017 for comparison of right- vs. left-hemispheric brain infarct) and 61.0% of patients with bi-hemispheric brain infarct (*p* = 0.700 for comparison of right- vs. bi-hemispheric brain infarct and *p* = 0.300 for comparison of left- vs. bi-hemispheric brain infarct).

### Exploratory Analyses: Association of HRV With Presence and Volume of Silent Brain Infarcts in the AF Group

The univariable OR for the association of HRV parameters with presence of SBI are presented in Supplementary Table 2. The OR was 1.65 (95% CI 1.08–2.53, *p* = 0.021) for HRVI <15 and 1.59 (95% CI 1.01–2.48, *p* = 0.046) for rMSSD. Other HRV parameters were not associated with presence of SBI. In the multivariable model, HRVI <15 was the only HRV parameter associated with the presence of SBI (OR 1.63, 95% CI 1.05–2.55, *p* = 0.031). For HRVI and presence of SBI, we found no interactions in any of the subgroups (Supplementary Table 3). None of the HRV parameters were associated with brain infarct volumes (Supplementary Table 4, Figure 2, right panel).

## Discussion

To the best of our knowledge, this is the first study to assess the relationship of HRV with SBI in AF patients. First, in the SR group, impaired HRVI was independently associated with the presence of SBI. Second, patients in the SR group with impaired HRVI had a higher volume of SBI. Moreover, patients with SBI in the right hemisphere showed more pronounced impairment of HRVI compared to patients with left-hemispheric brain infarcts. Exploratory analyses in the AF group showed that impaired HRVI was associated with the presence of SBI, but not with infarct volume.

In patients with AF, SBI have a similar impact on cognitive impairment as overt strokes (1) and are known to enhance the risk of subsequent overt strokes (4). The early identification of SBI or risk factors thereof may be important when risk stratifying AF patients. Few parameters (age, male sex and hypertension) have already been associated with SBI (20). However, the detected SBI in the NOMAS study were mostly small and subcortical (20) and only 4.2% of the participants had a history of AF. Moreover, although higher AF burden has been associated with an increasing risk of stroke (21), its association with SBI warrants further investigation.

Multiple studies have shown that patients with overt stroke show changes of HRV (22–26) that are mainly characterized by the predominance of sympathetic activity (22). Numerous studies suggest that HRV is impaired in stroke patients when compared to controls (23, 25, 26). Furthermore, HRV parameters may have the capacity to predict incident stroke (23, 25, 26). HRV measures can also be used as biomarkers for post-stroke outcomes such as mortality, complications and functionality (9, 27). However, all above mentioned studies have exclusively included SR patients. Only two studies included AF patients. The first study assessed HRV in stroke patients with permanent AF (*n* = 173) to predict overt stroke (28). The second study tested the association of HRV with 3-month post-stroke outcome and included a proportion of AF patients (*n* = 77) (29). Although HRV has already been thoroughly investigated in patients with overt strokes (22–27, 29), no prior investigation has assessed the association of HRV with SBI in AF patients.

In this first study assessing the association of HRV and SBI in patients with AF, we focused our analysis on the SR group, because HRV analysis is very well-established during SR (8), but has not been commonly used during AF so far. From a pathophysiological perspective, the meaning of HRV parameters measured during AF is less well-understood. It is questionable whether HRV calculated from AF recordings directly quantifies autonomic function. However, also in AF, HRV may be modulated by autonomic factors, for example on the level of the AV node (30). Therefore, we also performed exploratory analyses in the AF group to gain more information on the diagnostic meaning of HRV in AF, which might be a potential basis for further research.

HRVI, a geometrical HRV parameter (8), was independently associated with the presence of SBI. Impaired HRVI indicates imbalance of the autonomic nervous system, and is a predictor of adverse events such as malignant arrhythmias and mortality (31–33). Of note, other HRV parameter (including various time and frequency domain measures) were not associated with the presence of SBI in our study. An explanation may be that HRVI can be robustly calculated without manual editing of the RR interval series in a highly reproducible way and is highly insensitive to artifacts and ectopic beats (34).

In our study, patients with impaired HRVI (assessed during SR) had a higher volume of SBI. A potential mechanism might be that a larger involvement of the infarct area itself contributes to a more prominent impairment autonomic function. However, in the AF group, volumes of SBI did not differ when stratified by HRVI. This shows that analysis of HRV during AF may be less useful for risk stratification. Currently, it remains unclear how assessment of the ANS can be optimized in AF patients.

Furthermore, patients with right-hemispheric brain infarct showed a more pronounced impairment of HRVI compared to left-hemispheric brain infarcts. This finding is in line with previous studies showing that overt strokes in the right hemisphere lead to a derangement of HRV as the right hemisphere, especially the insular region, plays a major role in cardiac autonomic control (27, 35, 36). Our findings confirm that the severity of cardiac autonomic impairment might in part depend on the localization of a stroke.

The directionality of association between impairment of HRV and overt stroke is still unknown. On the one hand, the association could potentially originate from changes of autonomic control directly induced by the stroke, i.e., a complication of stroke (7). On the other hand, the association of HRV and stroke could also be explained by sharing the same cardiovascular risk factors. So far, the exact underlying pathways and mechanisms remain to be determined. Of note, it is unknown if SBI are associated with similar impairment of HRV as overt strokes. In our study, patients with a history of stroke/TIA were excluded (Figure 1). However, when comparing patients with a history of stroke/TIA from the Swiss-AF cohort study (*n* = 337) and patients with a SBI from our analysis (*n* = 194), HRV parameters were similar (detailed data not shown).

Strengths of our study are the large sample size of well-treated patients with AF who are comprehensively characterized. Limitations of our study are the cross-sectional design which precludes to assess the directionality of effect between impairment of HRV and SBI. Further, we did not perform 24-h Holter-ECG recordings. Therefore, our results are only valid for 5-min ECG recordings and we cannot judge the association between HRV measures calculated from long-term recordings and SBI. This is particularly relevant for HRVI, which is predominantly established in long-term ECG recordings so far. Further, as comprehensively discussed above, we are aware that HRV during AF may not have the same meaning as in SR. Finally, the generalizability to patients without AF or other population groups remains to be determined. Further studies are needed to validate our observations and cut-offs and clarify its clinical application.

In conclusion, autonomic dysfunction (assessed during SR) is independently associated with the presence and volume of SBI in patients with AF. HRV analysis may be a simple screening tool that enables clinicians to identify patients with an increased risk of SBI. Identified high risk patients may undergo bMRI, and in case SBI are detected, these patients may benefit from intensified treatment beyond standard oral anticoagulation, rhythm control as well as better control of comorbidities such as hypertension and diabetes. However, no study has proven the power of intensified treatment of AF and comorbidities on the occurrence of new brain lesions in AF patients.

## Data Availability Statement

Due to restrictions by the ethical committee data is not publicly available. Requests to access the datasets should be directed to christine.meyerzuern@usb.ch.

## Ethics Statement

The studies involving human participants were reviewed and approved by Ethics commitee University of Basel, Switzerland and all local ethics committees at the study sites. The patients/participants provided their written informed consent to participate in this study.

## Author Contributions

PH, CSZ, SO, and MK contributed to conception and design of the study. CEi and VS organized the ECG database. SB, SA, PK, and PM organized the clinical database. PH, CSZ, and MC performed the statistical analysis. PH wrote the first draft of the manuscript. CSZ, JW, TS, and MK wrote sections of the manuscript. SP, AB, KR, CEk, J-MV, JB, GM, LB, CS, DC, and SO gave intellectual input for manuscript writing. All authors contributed to the article and approved the submitted version.

## Conflict of Interest

CSZ reports a research grant from Medtronic and honoraria from Vifor Pharma and Novartis. MK has received grants from the Swiss National Science Foundation, the Swiss Heart Foundation, Daiichi-Sankyo, Bayer, and Pfizer-BMS and lecture/consulting fees from Daiichi-Sankyo, Boehringer Ingelheim, Bayer, Pfizer-BMS, AstraZeneca, Sanofi-Aventis, Novartis, MSD, Medtronic, Boston Scientific, St. Jude Medical, Biotronik, Sorin, Zoll and Biosense Webster. PK is supported by the University of Basel, the Mach-Gaensslen foundation and the Bangerter-Rhyner foundation. SP received research grants from Bristol-Myers Squibb/Pfizer, Daiichi Sankyo, and speakers' honoraria and consulting fees from Bayer, Boehringer Ingelheim, Bristol-Myers Squibb/Pfizer, Daiichi Sankyo and Werfen. JW is CEO of the Medical Image Analysis Center, Basel; has served on advisory boards for Actelion, Biogen, Genzyme-Sanofi, Novartis, Roche, and the Guthy Jackson Charitable Foundation; has received research grants from Novartis; has received speaker honoraria from Bayer, Biogen, Genzyme, Novartis, and Teva. The remaining authors declare that the research was conducted in the absence of any commercial or financial relationships that could be construed as a potential conflict of interest.
