# Supplementary material for: Association of Heart Rate Variability With Silent Brain Infarcts in Patients With Atrial Fibrillation
Source: Front Cardiovasc Med. 2021 May 21;8:684461. doi: 10.3389/fcvm.2021.684461 (PMC8175975; doi:10.3389/fcvm.2021.684461)
Supplement: Supplementary file 1 [file Data_Sheet_1.docx]

**SUPPLEMENTARY MATERIAL**

**Association of heart rate variability with silent brain infarcts in patients with atrial fibrillation**

Peter Hämmerle MD, Christian Eick MD, Sven Poli MD, Steffen Blum MD PhD, Vincent Schlageter PhD, Axel Bauer MD, Konstantinos D Rizas MD, Ceylan Eken PhD, Michael Coslovsky PhD, Stefanie Aeschbacher PhD, Philipp Krisai MD, Pascal Meyre, MD PhD, Jens Wuerfel MD, Tim Sinnecker MD, Jean-Marc Vesin PhD, Jürg H. Beer MD, Giorgio Moschovitis MD, Leo H. Bonati MD, Christian Sticherling MD, David Conen MD, Stefan Osswald MD, Michael Kühne MD and Christine S Zuern MD; on behalf of the Swiss-AF Study Investigators***.**

*** Swiss-AF investigators**

University Hospital Basel and Basel University: Stefanie Aeschbacher, Chloe Auberson, Steffen Blum, Leo Bonati, Selinda Ceylan, David Conen, Simone Doerpfeld, Marc Girod, Peter Hämmerle, Philipp Krisai, Michael Kühne, Christine Meyer-Zürn, Pascal Meyre, Andreas U. Monsch, Christian Müller, Stefan Osswald, , Philipp Reddiess, Javier Ruperti Repilado, Anne Springer, Fabienne Steiner, Christian Sticherling, Thomas Szucs, Gian Voellmin, Leon Zwimpfer. Principal Investigator: Stefan Osswald; Local Principal Investigator: Michael Kühne

University Hospital Bern: Faculty: Drahomir Aujesky, Urs Fischer, Juerg Fuhrer, Laurent Roten, Simon Jung, Heinrich Mattle; Research fellows: Luise Adam, Carole Elodie Aubert, Martin Feller, Claudio Schneider, Axel Loewe, Elisavet Moutzouri; Study nurses: Tanja Flückiger, Cindy Groen, Damiana Rakovic, Rylana Wenger, Lukas Ehrsam, Alexandra Nuoffer, Nathalie Schwab. Local Principal Investigator: Nicolas Rodondi

Stadtspital Triemli Zurich: Christopher Beynon, Roger Dillier, Michèle Deubelbeiss,  Franz Eberli, Christine Franzini, Isabel Juchli, Claudia Liedtke, Jacqueline Nadler, Thayze Obst, Noreen Tynan, Xiaoye Schneider, Katrin Studerus, Dominik Weishaupt. Local Principal Investigator: Andreas Müller

Kantonspital Baden: Simone Fontana, Silke Kuest, Karin Scheuch, Denise Hischier, Nicole Bonetti, Alexandra Grau, Jonas Villinger, Eva Laube, Philipp Baumgartner, Mark Filipovic, Marcel Frick, Giulia Montrasio, Stefanie Leuenberger, Franziska Rutz. Local Principal Investigator: Jürg-Hans Beer

Cardiocentro Lugano: Angelo Auricchio, Adriana Anesini, Cristina Camporini, Giulio Conte, Maria Luce Caputo, Francois Regoli. Local Principal Investigator: Tiziano Moccetti

Kantonsspital St. Gallen: Roman Brenner, David Altmann, Michaela Gemperle. Local Principal Investigator: Peter Ammann

Hôpital Cantonal Fribourg: Mathieu Firmann, Sandrine Foucras. Local Principal Investigator: Daniel Hayoz

Luzerner Kantonsspital: Benjamin Berte, Virgina Justi, Frauke Kellner-Weldon, Brigitta Mehmann, , Myriam Roth, Andrea Ruckli-Kaeppeli, Ian Russi, Kai Schmidt, Mabelle Young, Melanie Zbinden. Local Principal Investigator: Richard Kobza

Ente Ospedaliero Cantonale Lugano:  Jane Frangi-Kultalahti, Anica Pin, Luisa Vicari Local Principal Investigator: Giorgio Moschovitis

University Hospital Geneva: Georg Ehret, Hervé Gallet, Elise Guillermet, Francois Lazeyras, Karl-Olof Lovblad, Patrick Perret, Philippe Tavel, Cheryl Teres. Local Principal Investigator: Dipen Shah

University Hospital Lausanne: Nathalie Lauriers, Marie Méan, Sandrine Salzmann. Local Principal Investigator: Jürg Schläpfer

Bürgerspital Solothurn: Andrea Grêt, Jan Novak, Sandra Vitelli. Local Principal Investigator: Frank-Peter Stephan

Ente Ospedaliero Cantonale Bellinzona: Jane Frangi-Kultalahti, Augusto Gallino. Local Principal Investigator: Marcello Di Valentino

University of Zurich/University Hospital Zurich: Fabienne Witassek, Matthias Schwenkglenks.

Medical Image Analysis Center AG Basel: Jens Würfel (Head), Anna Altermatt, Michael Amann, Petra Huber, Esther Ruberte, Tim Sinnecker, Vanessa Zuber.

Clinical Trial Unit Basel: Michael Coslovsky (Head), Pascal Benkert, Gilles Dutilh, Milica Markovic, Patrick Simon

Schiller AG Baar: Ramun Schmid

**Suppl. Table 1** Parameters of heart rate variability in patients stratified by baseline rhythm

**Suppl. Table 2** Association of heart rate variability and presence of silent brain infarcts in the atrial fibrillation group (exploratory analysis)

**Suppl. Table 3** Association of heart rate variability and silent brain infarct volume in the atrial fibrillation group (exploratory analysis)

**Suppl. Table 4** Association between heart rate variability triangular index and silent brain infarcts: subgroup analyses in the atrial fibrillation group (exploratory analysis)

**Suppl. Table 1** Parameters of heart rate variability in patients stratified by baseline rhythm

| **HRV parameter** | **Sinus rhythm group**  **(N=816)** | **Atrial fibrillation group**  **(N=542)** | **p-value** |
| --- | --- | --- | --- |
| HRVI | 14.6 (11.8-18.1) | 15.6 (12.9-18.9) | <0.001* |
| SDNN (ms) | 85.9 (44.7-145.1) | 100.5 (78.1-134.8) | <0.001* |
| MHR (bpm) | 85.8 (63.5-142.2) | 125.9 (87.5-151.5) | <0.001* |
| RMSSD (ms) | 35.30 (20.0-46.4) | 54.2 (41.0-74.1) | <0.001* |
| 5-minute total power | 3.38±0.79 | 3.67±0.29 | <0.001+ |
| HF | 2.79±0.77 | 3.07±0.34 | <0.001+ |
| LF | 3.22±0.83 | 3.51±0.33 | <0.001+ |
| VLF | 3.11±0.86 | 3.26±0.65 | <0.001+ |

Data are medians and interquartile ranges or means and standard deviations. P-value compares sinus rhythm and atrial fibrillation groups and was obtained from Mann-Whitney-Test* or from Student`s t-test+. HF = high frequency. HRV = heart rate variability. HRVI = heart rate variability triangular index. LF = low frequency. MHR = mean heart rate. rMSSD = root mean square root of successive differences of normal-to-normal intervals. SDNN = standard deviation of the normal-to-normal intervals. VLF = very low frequency. Frequency measures of HRV were log transformed.

**Suppl. Table 2** Association of heart rate variability and presence of silent brain infarcts in the atrial fibrillation group (exploratory analysis)

| **HRV parameter** | **Univariable model**  OR (95% CI) | **p-value*** | **Multivariable model**  OR (95% CI) | **p-value*** |
| --- | --- | --- | --- | --- |
| **Time domain measures** | | | | |
| HRVI<15 | 1.65 (1.08-2.53) | 0.021 | 1.63 (1.05-2.55) | 0.031 |
| SDNN<70ms | 1.45 (0.84-2.50) | 0.185 | 1.52 (0.86-2.68) | 0.148 |
| rMSSD<42ms | 1.59 (1.01-2.48) | 0.046 | 1.52 (0.95-2.48) | 0.078 |
| MHR>80bpm | 0.83 (0.45-1.51) | 0.530 | 0.76 (0.41-1.41) | 0.381 |
| **Frequency domain measures**^†^ | | | | |
| 5-min total power | 1.09 (0.52-2.28) | 0.814 | 1.08 (0.50-2.43) | 0.848 |
| LF | 0.97 (0.51-1.84) | 0.924 | 0.95 (0.49-1.86) | 0.886 |
| HF | 1.34 (0.71-2.54) | 0.363 | 1.41 (0.71-2.76) | 0.328 |
| VLF | 0.98 (0.70-1.39) | 0.927 | 0.99 (0.70-1.41) | 0.966 |

Data are odds ratios (95% confidence intervals). *p-values were based on logistic regression models. ^†^ Frequency domain measures of HRV have been log-transformed^.^ Study center was included as random intercept. Multivariable model was adjusted for age, sex, systolic blood pressure, history of hypertension, history of diabetes, history of heart failure, history of pulmonary vein isolation, prior myocardial infarction, prior major bleeding, intake of oral anticoagulation, antiarrhythmics and betablockers. HF = high frequency (0.15-0.4 Hz). HRV = heart rate variability. HRVI = heart rate variability triangular index. MHR = mean heart rate. LF = low frequency (0.04-0.15 Hz). OR = odds ratio. rMSSD = root mean square root of successive differences of normal-to-normal intervals. SDNN = standard deviation of the normal-to-normal intervals. VLF = very low frequency (≤0.04 Hz).

**Suppl. Table 3** Association of heart rate variability and silent brain infarct volume in the atrial fibrillation group (exploratory analysis)

| **Subgroup** | **No. of events/n** | **Odds ratio** | **95% CI** | **p-value** | **p-interaction** |
| --- | --- | --- | --- | --- | --- |
| **Age** |  |  |  |  |  |
| Age<median | 42/271 | 2.19 | 1.12-4.28 | 0.022 | 0.443 |
| Age≥median | 66/271 | 1.29 | 0.74-2.24 | 0.378 |  |
| **Sex** |  |  |  |  |  |
| Male | 88/424 | 1.97 | 1.22-3.19 | 0.006 | 0.503 |
| Female | 20/118 | 0.71 | 0.25-2.00 | 0.513 |  |
| **AF type** |  |  |  |  |  |
| Paroxysmal | 8/66 | 0.79 | 0.17-3.63 | 0.764 |  |
| Persistent | 33/167 | 1.96 | 0.90-4.25 | 0.091 | 0.155 |
| Permanent | 67/309 | 1.65 | 0.96-2.85 | 0.071 |  |
| **History of diabetes** |  |  |  |  |  |
| Yes | 19/99 | 1.23 | 0.45-3.34 | 0.688 | 0.545 |
| No | 89/443 | 1.76 | 1.10-2.82 | 0.018 |  |
| **History of myocardial infarction** |  |  |  |  |  |
| Yes | 18/87 | 2.84 | 0.91-8.82 | 0.072 | 0.479 |
| No | 90/455 | 1.45 | 0.94-2.38 | 0.088 |  |
| **Intake of antiarrhythmic drugs (class Ic, II & III)** |  |  |  |  |  |
| Yes | 86/418 | 1.67 | 1.03-2.69 | 0.037 | 0.582 |
| No | 22/124 | 1.58 | 0.63-3.99 | 0.332 |  |
| **History of pulmonary vein isolation** |  |  |  |  |  |
| Yes | 5/38 | 4.00 | 0.57-28.0 | 0.163 | 0.945 |
| No | 103/504 | 1.55 | 1.00-2.40 | 0.049 |  |

Univariable analyses are presented. AF=atrial fibrillation. CI=confidence interval. No=number. n=number of patients included in the subgroup.

**Suppl. Table 4** Association between heart rate variability triangular index and silent brain infarcts: subgroup analyses in the atrial fibrillation group (exploratory analysis)

| **HRV parameter** | **Univariable model**  β (95% CI) | **p-value*** | **Multivariable model**  β (95% CI) | **p-value*** |
| --- | --- | --- | --- | --- |
| **Time domain measures** | | | | |
| HRVI<15 | 0.09 (-0.21; 0.38) | 0.562 | 0.07 (-0.27; 0.41) | 0.668 |
| SDNN<70ms | -0.01 (-0.38; 0.37) | 0.970 | 0.02 (-0.43; 0.43) | 0.991 |
| rMSSD<42ms | 0.04 (-0.28; 0.35) | 0.825 | 0.06 (-0.29; 0.40) | 0.750 |
| MHR>80bpm | 0.03 (-0.40; 0.46) | 0.897 | 0.04 (-0.43; 0.51) | 0.877 |
| **Frequency domain measures**^†^ | | | | |
| 5-min total power | 0.37 (-0.15; 0.89) | 0.167 | 0.31 (-0.26; 0.88) | 0.282 |
| LF | 0.25 (-0.21; 0.70) | 0.284 | 0.22 (-0.27; 0.71) | 0.379 |
| HF | 0.31 (-0.14; 0.77) | 0.173 | 0.24 (-0.26; 0.74) | 0.340 |
| VLF | 0.08 (-0.15; 0.31) | 0.504 | 0.07 (-0.17; 0.31) | 0.565 |

Data are beta-coefficients (β) (95% confidence intervals [CI]). Brain infarct volumes were log-transformed.*p-values were based on linear regression models. ^†^ Frequency domain measures of HRV have been log-transformed. Study center was included as random intercept. Multivariable model was adjusted for age, sex, systolic blood pressure, history of hypertension, history of diabetes, history of heart failure, history of pulmonary vein isolation, prior myocardial infarction, prior major bleeding, intake of oral anticoagulation, antiarrhythmics and betablockers. HF = high frequency (0.15-0.4 Hz). HRV = heart rate variability. HRVI = heart rate variability triangular index. MHR = mean heart rate. LF = low frequency (0.04-0.15 Hz). rMSSD = root mean square root of successive differences of normal-to-normal intervals. SDNN = standard deviation of the normal-to-normal intervals. VLF = very low frequency (≤0.04 Hz).
